# Reduced Resting State Neural Activity in the Right Orbital Part of Middle Frontal Gyrus in Anxious Depression

**DOI:** 10.3389/fpsyt.2019.00994

**Published:** 2020-01-22

**Authors:** Peng Zhao, Rui Yan, Xinyi Wang, Jiting Geng, Mohammad Ridwan Chattun, Qiang Wang, Zhijian Yao, Qing Lu

**Affiliations:** ^1^ Department of Psychiatry, The Affiliated Brain Hospital of Nanjing Medical University, Nanjing, China; ^2^ Department of Medical Psychology, Nanjing Drum Tower Hospital, The Affiliated Hospital of Nanjing University Medical School, Nanjing, China; ^3^ School of Biological Sciences & Medical Engineering, Southeast University, Nanjing, China; ^4^ Child Development and Learning Science, Key Laboratory of Ministry of Education, Nanjing, China; ^5^ Department of Psychiatry, Hangzhou Seventh People's Hospital, Hangzhou, China; ^6^ Nanjing Brain Hospital, Medical School of Nanjing University, Nanjing, China

**Keywords:** anxious depression, resting-state functional magnetic resonance, amplitude of low-frequency fluctuation, functional connectivity, right orbital part of middle frontal gyrus

## Abstract

**Background:**

Anxious depression (AD), which is generally recognized as a common clinical subtype of major depressive disorder (MDD), holds distinctive features compared with unanxious depression (UAD). However, the neural mechanism of AD still remains unrevealed. To give insight to it, we compared resting-state functional magnetic resonance amplitude of low-frequency fluctuation (ALFF) and functional connectivity (FC) between AD and UAD patients.

**Method:**

The data were collected from 60 AD patients, 38 UAD patients, and 60 matched healthy controls. The ALFF and seed-based FC were examined. Pearson correlations were computed between ALFF/FC and clinical measures.

**Results:**

In Comparison with the UAD group, the ALFF value of the right orbital part of middle frontal gyrus (RO-MFG) decreased in AD group. Specifically, the ALFF values of the RO-MFG were negatively correlated with retardation factor scores in AD group (r = -0.376, *p* = 0.003).

**Conclusions:**

AD patients exhibited disturbed intrinsic brain function compared with UAD patients. The decreased activity of the RO-MFG is indicative of the alterations involved in the neural basis of AD.

## Introduction

Major depressive disorder (MDD), which ranks second among the leading cause of disability in the world (1), has both high incidence and clinical severity (2). A significant factor which enhanced the severity of MDD is its high comorbidity with anxiety disorders (2–5). As a common clinical subtype of MDD, anxious depression (AD) has more severe depressive symptoms, somatic symptoms, and frequent episodes as well as a higher risk of suicidal tendency compared with unanxious depression (UAD) (6–9). In addition to having a lower response to antidepressants and more drug side effects, patients with AD usually take twice as long to recover from episode (9–13). These combined downsides motivated us to further uncover the neural mechanisms behind AD.

While AD is common in clinical practice, an in-depth understanding toward its neuromechanism is limited. Structural magnetic resonance imaging studies found that AD patients had brain structural abnormalities in the cortical-limbic circuit, which were involved in emotion regulation (14–16). Similar brain abnormalities also have been found in functional magnetic resonance imaging (fMRI) studies (17–21). For instance, hyperactivity was found in dorsal anterior cingulated cortex (ACC), posterior cingulated cortex, and prefrontal cortex supplementary motor area when AD patient was performing the Preparing to Overcome Prepotency task (17). In the emotion induction task study, AD patients had lower activity of the bilateral anterior lateral prefrontal cortices compared to UAD patients (19). The inconsistent results in the two studies were due to the difference of methodology, such as the different tasks used in task-related fMRI. Previous studies showed that AD symptoms was unlikely to result from a single brain region and the neural mechanism of AD encompassed a number of distributed networks (22).

Resting state fMRI (rs-fMRI) does not require patients to perform any task and is easier to implement in clinical practice (23). The amplitude of low-frequency fluctuation (ALFF) reflects the strength of spontaneous neural activity of a voxel (24). Functional connectivity (FC) indicates the inter-regional temporal correlation between predefined seed regions and related functional regions and the strength of the connection relationship (25). In the past, both of these methods have been used to measure the spontaneous fluctuations of blood oxygenation level dependent fMRI signal intensity in psychiatric diseases such as MDD (26), Alzheimer's disease (27), and attention deficit hyperactivity disorder (28).

So far, to the best of our knowledge, only two rs-fMRI studies elucidated the characteristics of brain functional changes in AD. The first study of elderly subjects found that patients with AD manifested distinct features of connectivity in default mode network (DMN) when compared with the patients with UAD (20). There was significantly increased functional connectivity (FC) in the posterior regions of the DMN and decreased FC in the anterior regions of the DMN in AD patients. However, the elderly subjects may have its unique brain functional characteristics, which could not be transferred to another AD. In the second study, AD patients displayed increased activity in the right dorsal ACC and the right ventral ACC as well as a decreased activity in the bilateral lingual gyrus in contrast to remitted depression patients and healthy controls (HC) (21). However, since the patients of the control group were remitted depression patients, the results could not effectively distinguish between AD and UAD.

Depression and anxiety are conventionally conceptualized as two independent disorders. Since AD could be seen as MDD plus the presence of at least co-morbid anxiety disorder (22, 29), separate studies on both MDD and anxious disorder could provide hints on the inner neural mechanism of AD. According to the recent investigations (30, 31), dysfunctional neural systems pertaining to MDD mainly include the subcortical systems implicated in emotion and reward processing, for e.g., amygdale and ventral striatum. Moreover, there were aberrancies in medial prefrontal cortex (MPFC), orbitofrontal cortex (OFC), ACC regions involved with automatic regulation of emotion, and lateral prefrontal cortical (LPFC) systems involved with voluntary regulation of emotion in MDD patients. A meta-analysis of fMRI in MDD showed increased activity in neural systems that produce emotion and automatic regulate emotion (amygdale and MPFC) as well as reduced activity in neural systems that voluntary regulate emotion (32). Another meta-analysis of resting-state FC suggested that MDD was characterized by hyperconnectivities within DMN and hypoconnectivities within the frontoparietal network (33). However, the above studies are associated with only MDD and AD could potentially have its unique characteristics.

Studies associated with anxiety disorder have revealed that the brain regions are overlapped with most of the regions involved in MDD namely the amygdale, MPFC, OFC, ACC, and LPFC (34, 35). In addition, the brain network dysfunction related to anxiety disorder were similar to those of MDD (e.g., decreased functioning of the frontoparietal network) (36). However, patients with anxiety disorder also had different functional network dysfunction compared with MDD patients (e.g., decreased or under-active functioning of DMN) (36).

In order to further shed light on the underlying neuropathological mechanisms of AD, we investigated the different brain dysfunctions between AD and UAD. Based on pre-reviewed studies, we made two hypotheses: 1) firstly, we hypothesized that AD would exhibit abnormal activities in the overlapping brain regions between MDD and anxious disorder, which specifically include the regions involved in emotion and reward processing, automatic regulation of emotion, and voluntary regulation of emotion. 2) Second, we expect to observe reduced neural activities in brain regions that automatic regulate emotion in patients with AD compared with UAD during the resting state.

## Methods

### Participants

The participants in this research consisted of patients and HCs. One hundred and eleven treatment-naive patients with first-episode MDD were recruited from the Department of Psychiatry of the Affiliated Nanjing Brain Hospital of Nanjing Medical University and the Department of Medical Psychology of the Affiliated Drum Tower Hospital of Medical School of Nanjing University from September 2011 to October 2017. Patients were selected based on the following inclusion criteria: 1) a diagnosis of MDD according to the criteria of the fourth edition of the Diagnostic and Statistical Manual of Mental Disorders and by the Mini International Neuropsychiatric Interview (MINI) (37); 2) a total score higher than 17 on the 17-item Hamilton Rating Scale for Depression (HRSD); 3) aged between 18 and 55 years; 4) no current and/or prior use of antidepressants and treatment with cognitive behavioral therapy or physiotherapy; 5) no family history of mental illness in first-degree relatives; 6) no psychotic symptoms; 7) no comorbidity with other major psychiatric illness or neurological illness such as schizophrenia, bipolar disorder, developmental delay, personality disorder, or substance abuse; 8) no evidence of serious medical diseases or organic brain disorders.

Sixty-two well-matched HCs were enrolled from the local community *via* advertisements. HCs were assessed with MINI in order to confirm the absence of a history of mental illness. All participants were right-handed Han Chinese. Exclusion criteria for all participants were as follows: 1) age under 18 or above 55 years; 2) a family history of psychiatric disorder in first-degree relatives; 3) serious medical or neurological disorders; 4) substance abuse or dependence; 5) current pregnancy or breastfeeding; 6) MRI contraindications.

Before starting the experiment, the detailed procedure was explained to the participants and informed consents, which were in handwritten forms as approved by the Research Ethics Review Board of Affiliated Nanjing Brain Hospital of Nanjing Medical University, were collected.

Since the current study is related to the differences between AD and UAD, their specific definition were as follows: AD was defined as MDD with severe anxiety symptoms (anxiety/somatization factor score ≥7 in the 17-item HRSD) (6, 7, 11, 38); UAD was defined as MDD with low levels of anxiety (anxiety/somatization factor score of <7, which is the opposite of AD) (6, 7, 11, 38). The anxiety/somatization factor score include general somatic symptoms, gastrointestinal somatic symptoms, hypochondriasis, insight, psychic anxiety, and somatic anxiety (38). In order to obtain more detailed clinical information, each patient was assessed with Hamilton Anxiety Scale (HAMA).

### Magnetic Resonance Imaging Data Acquisition

On the day of enrollment, MRI image data was obtained by a 3-Tesla Siemens Verio scanner of an eight-channel radio frequency coil at the Affiliated Brain Hospital of Nanjing Medical University. All subjects' heads were positioned in a birdcage coil. Earplugs were utilized to reduce scanning noise. The participants were instructed to relax, close their eyes and not think of anything during the scan. In this study, The parameters for T1 anatomic axial imaging and rs-fMRI set is similar to the parameters in Yan et al. (39).

### Data Preprocessing

Data Processing Assistant for Resting-State fMRI (DPARSF) toolbox (http://www.restfmri.net/forum/DPARSF) was used to preprocess the image. MIRcroN (http://www.mricro.com) was employed to conduct image format transduction. In order to stabilize magnetization and adapt the participants to the scanner environment, the first 6 volumes were removed from original data. The residual 127 volumes were slice timed and the head-motion was realigned. With the aim of minimizing the influence of head displacement, 10 patients with more than 2 mm movement in any direction and 2 of angular motion during the fMRI scan were excluded from the study. An estimation of head motion at each time point was calculated as frame-wise displacement (FD) using six displacements from the rigid body motion correction procedure. The residual data were normalized in the Montreal Neurological Institute (MNI) space, re-sampled with 3×3×3 mm^3^ resolution, and smoothed with Gaussian kernel (full-width at half maximum = 4 mm). After smoothing, the data underwent temporal filtering (0.01–0.08 Hz) and linear detrending to reduce the influence of the low-frequency drift and high-frequency noise. Five participants (two HCs and three patients) were excluded from the experiment due to abnormal brain signals. Finally, 98 patients (60 AD and 38 UAD) and 60 HCs were included for ALFF and FC analysis in DPARSF.

### Amplitude of Low-Frequency Fluctuation Analyses

ALFF was calculated with the DPARSF toolbox. Fast Fourier transform was applied on filtered time series of each voxel to transform it to frequency domain. The averaged square root across 0.01–0.08 Hz at each voxel was taken as the ALFF measurement (24).

### Functional Connectivity Analyses

Regions with significant ALFF differences between the two depression groups were recognized as regions of interest (ROI). The ROIs were represented by a sphere with a 6-mm radius. The FC between the ROIs and the whole brain was computed using the DPARSF toolbox. A time series of ROIs and whole brain were extracted and averaged across all time series. The nuisance covariates, including cerebrospinal fluid signals, global mean signals, white matter signals and head motion parameters, were regressed out from each region. In order to eliminate individual position difference, individual images were normalized into a standard template. Pearson's correlation coefficients between ROIs and the whole brain regions represented the strength of the FC. The correlation coefficients were converted to z-scores with Fisher's r-to-z transform for further analysis.

### Statistical Analysis

The distribution of gender, years of education, and age among three groups were analyzed. Chi-square test and one-way analysis of variance (ANOVA) were calculated in SPSS (version 19.0, SPSS, Chicago, IL, USA). Non-parametric tests were employed when the variance was heterogeneous. Duration of illness and HRSD-17 scores between AD and UAD groups were compared using two-sample t-test. Significance was set at *p* < 0.05 and all tests were two-tailed. ANOVA was conducted to compare whole brain ALFF and FC using Resting-State fMRI Data Analysis Toolkit (REST) software (http://www.restfmri.net/forum/REST). *Post hoc* t-test was then utilized to confirm groups' difference. In order to avoid undetectable influences, age, gender, and years of education were taken as covariates. The corrected threshold was determined using the AlphaSim program, with the threshold set at *p* < 0.001 and a cluster size > 17 voxels in the ANOVA analysis and a cluster size > 1 voxel in *post hoc* t-test (http://afni.nimh.nih.gov/pub/dist/doc/manual/AlphaSim.pdf). The AlphaSim calculation was performed using REST software, which integrated the true smoothness kernel based on the statistical map and mask file. To investigate the relationship between ALFF, FC, and clinical features in depression in the two patients' groups, the ALFF and FC values that differed significantly between two depression groups were extracted and correlated with total score of HRSD, each item of the HRSD, HAMA, and disease duration. Bonferroni correction was used to solve multiple comparison problems. The alpha value was adjusted by dividing the regular alpha of 0.05 by the number of tests performed, resulting in a Bonferroni corrected *p* value of 0.05/8 (0.0063).

## Results

### Demographic and Psychometric Data

Clinical, demographic characteristics and the FD of the participants are displayed in **Table 1**. Gender, age, education level, and FD among the three groups did not differ significantly. There was no significant difference in the duration of illness between AD and UAD group. The AD group had higher HRSD-17 scores, anxiety/somatization factor scores, and HAMA scores compared to the UAD group, while the scores of HRSD-17 scores minus anxiety/somatization factor scores showed no group differences.

**Table 1:** Demographic, clinical characteristics and frame-wise displacement of all subjects.

Variables (mean ± SD)	AD(n = 60)	NSD(n = 38)	HC(n = 60)	t/F	*p*-value
Sex (male/female)	28/32	19/19	34/26	1.233	0.540^#^
Age (years)	33.53 ± 8.59	31.50 ± 8.97	33.55 ± 9.21	0.757	0.471^*^
Education lever (years)	13.72 ± 2.96	14.13 ± 3.02	14.60 ± 1.72	4.044	0.132^∆^
Duration of illness (month)	7.45 ± 9.50	7.87+9.78		0.044	0.834**
HRSD-17	26.35 ± 4.78	20.87 ± 3.23		6.768	0.000**
Anxiety/somatization factor	9.25 ± 1.65	5.03 ± 1.05		15.454	0.000**
Weight factor	0.95 ± 0.89	0.76 ± 0.82		1.043	0.300**
Cognitive disturbance factor	4.77 ± 2.35	4.45 ± 2.09		0.684	0.495**
Diurnal variation factor	0.50 ± 0.72	0.53 ± 0.73		-0.175	0.861**
Retardation factor	8.17 ± 1.51	7.53 ± 1.84		1.878	0.063**
Sleep disturbance factor	3.93 ± 1.76	3.76 ± 2.06		0.436	0.664**
Hopelessness factor	5.25 ± 2.03	4.92 ± 2.57		0.668	0.507**
HRSD-17-anxiety/somatization Factor	17.10 ± 4.15	15.84 ± 3.16		1.567	0.120**
HAMA	25.72 ± 7.29	16.21 ± 3.93		8.360	0.000**
FD	0.11 ± 0.06	0.11 ± 0.07	0.11 ± 0.06	0.068	0.93*

SD, standard deviation; AD, anxious depression; UAD, unanxious depression; HC, healthy control; HRSD, Hamilton Depression Rating Scale; HAMA, Hamilton Anxiety Scale; FD, frame-wise displacement. ^#^ indicates p values for chi-square test. * indicates p values for one-way ANOVA. ^∆^ indicates p values for non-parametric test. ** indicates p values for two-sample t-tests.

### Amplitude of Low-Frequency Fluctuation: Group Differences

**Figure 1:**
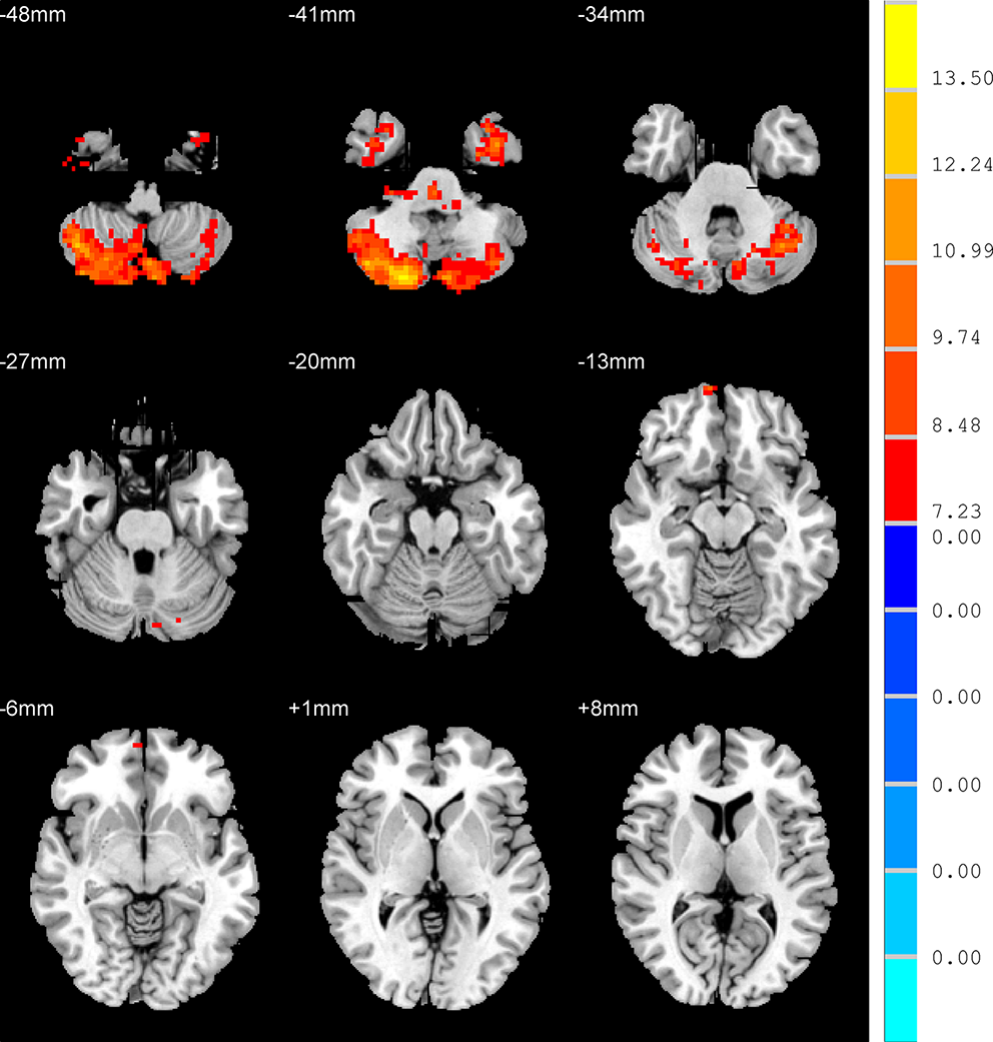
Brain regions show the differences in the amplitudes of low-frequency fluctuations among the three groups. The color bar signifies the F-value of the analysis of variance analysis with *p* < 0.001 and corrected for multiple comparisons using AlphaSim.

One-way ANOVA demonstrated that there were four regions with significantly different ALFF in the three groups, namely right inferior temporal gyrus, left inferior temporal gyrus, the right orbital part of middle frontal gyrus (RO-MFG), and right cerebelum crus2 (*p* < 0.001, k > 17 voxels, *p* < 0.05 corrected for multiple comparisons with AlphaSim) (**Figure 1**, **Table 2**).

**Table 2:** Brain areas with amplitude of low-frequency fluctuation (ALFF) difference among all groups.

Brain areas (AAL)	Peak MNI coordinates			Voxels	F/t value
	x	y	z		
Three group			
R inferior temporal gyrus	39	−3	−39	56	9.111^a^
R orbital part of middle frontal gyrus	9	66	−12	18	9.782^a^
L inferior temporal gyrus	−33	12	−45	76	14.751^a^
R cerebelum crus2	39	−66	−45	1,224	14.590^a^
AD < UAD			
R orbital part of middle frontal gyrus	3	60	−6	2	−3.739^b^
AD < HC			
R cerebelum crus2	39	−66	−45	120	−3.931^b^
L cerebelum crus2	−39	−75	−42	12	−3.526^b^
R cerebelum crus1	30	−69	−36	11	−3.732^b^
UAD < HC			
R inferior temporal gyrus	33	9	−45	43	−4.074^b^
R cerebelum crus2	21	−84	−42	157	−4.089 ^b^
L cerebelum crus2	−9	−84	−27	12	−3.582^b^
L cerebelum crus1	−36	−63	−33	80	−3.953^b^
UAD > HC			
R orbital part of middle frontal gyrus	9	66	−9	4	3.689^b^

AAL, anatomical automatic labeling; MNI, Montreal Neurologic Institute; AD, anxious depression; UAD, unanxious depression; HC, healthy control; ALFF, amplitude of low-frequency fluctuation; L, left; R, right.

a The F statistical value.

b The t statistical value.

### Amplitude of Low-Frequency Fluctuation: Anxious Depression *Versus* Unanxious Depression Patients

**Figure 2:**
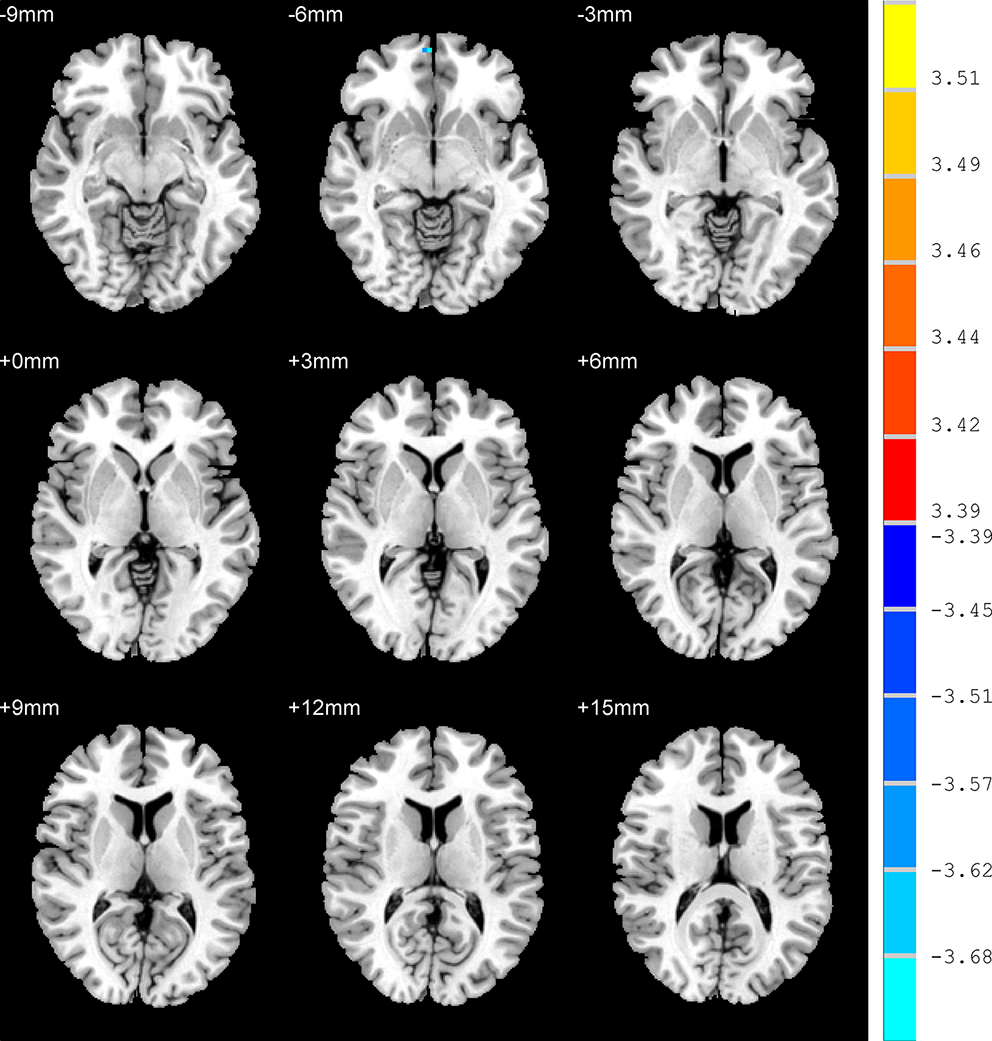
Brain regions show the differences in the amplitudes of low-frequency fluctuations between anxious depression group and unanxious depression group. The color bar signifies the t-value of the independent *t*-tests between the two groups with *p* < 0.001 and corrected for multiple comparisons using AlphaSim.

In contrast to the UAD patients, the AD patients displayed significantly lower ALFF values in the RO-MFG (*p* < 0.001, k > 1 voxel, *p* < 0.05 corrected for multiple comparisons using AlphaSim) (**Figure 2**, **Table 2**)

### Amplitude of Low-Frequency Fluctuation: Anxious Depression Patients *Versus* Healthy Control

**Figure 3:**
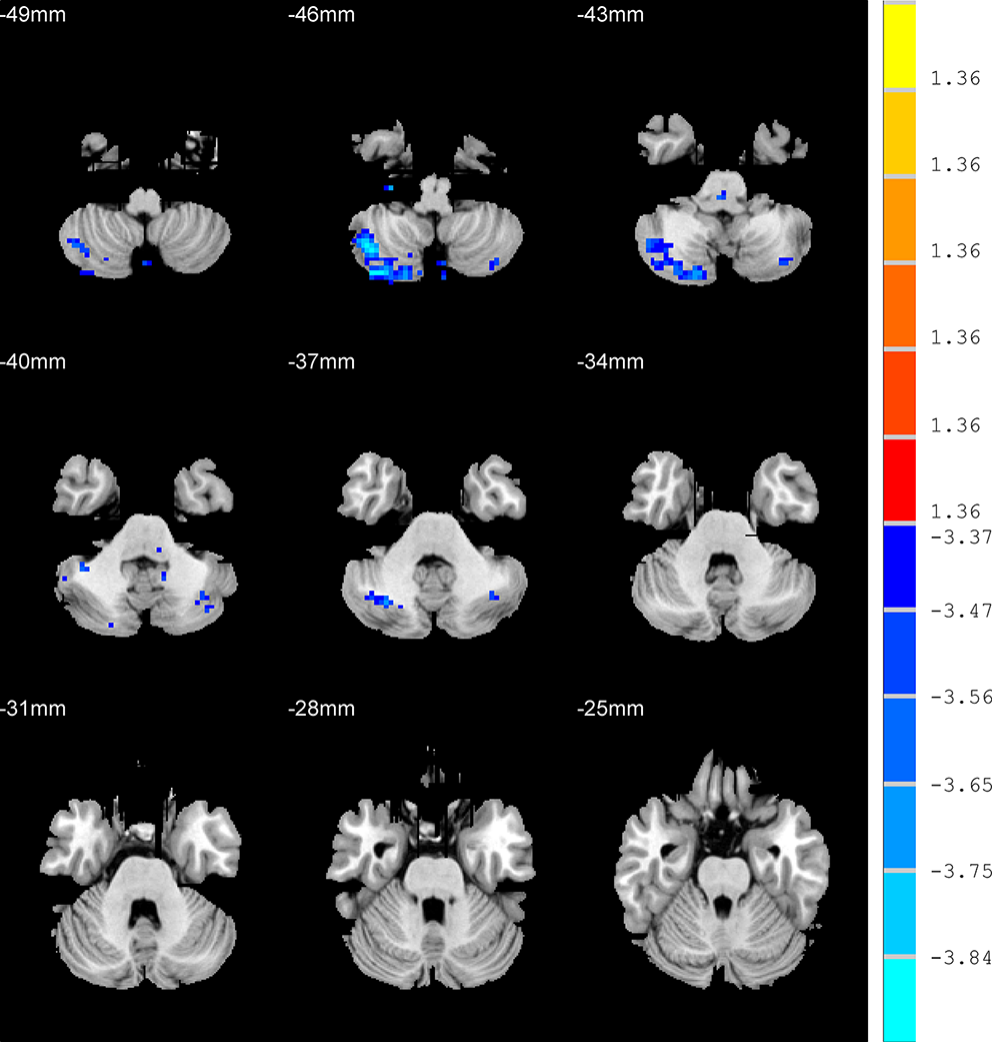
Brain regions show the differences in the amplitudes of low-frequency fluctuations between anxious depression group and healthy control group. The color bar signifies the t-value of the independent *t*-tests between the two groups with *p* < 0.001 and corrected for multiple comparisons using AlphaSim.

Compared with HC group, the AD patients showed lower ALFF values in the right cerebelum crus2, right cerebelum crus1, and left cerebelum crus2 (*p* < 0.001, k > 1 voxel, *p* < 0.05 corrected for multiple comparisons using AlphaSim) (**Figure 3**, **Table 2**).

### Amplitude of Low-Frequency Fluctuation: Unanxious Depression Patients *Versus* Healthy Control

**Figure 4:**
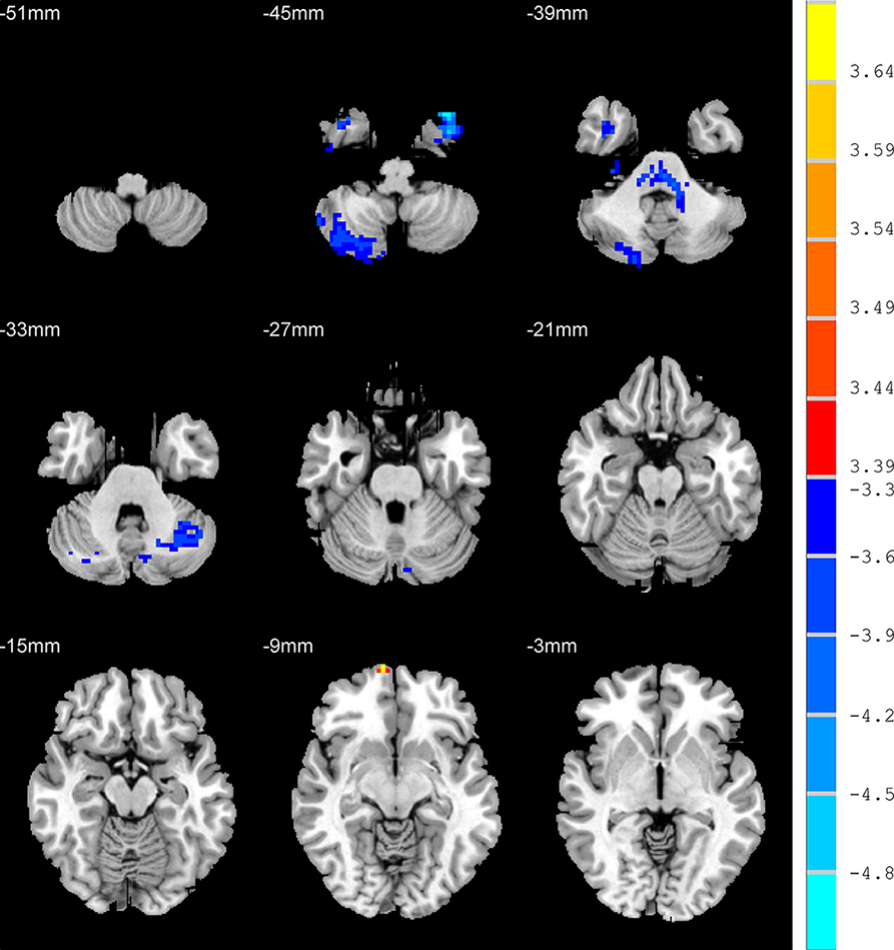
Brain regions show the differences in the amplitudes of low-frequency fluctuations between unanxious depression group and healthy control group. The color bar signifies the t-value of the independent *t*-tests between the two groups with *p* < 0.001 and corrected for multiple comparisons using AlphaSim.

Relative to HC group, the AD patients exhibited higher ALFF values in the RO-MFG, and lower values in right inferior temporal gyrus, right cerebelum crus2, left cerebelum crus1, and left cerebelum crus2 (*p* < 0.001, k > 1 voxel, *p* < 0.05 corrected for multiple comparisons using AlphaSim) (**Figure 4**, **Table 2**).

### Exploratory Correlational Analysis Between the Amplitude of Low-Frequency Fluctuation and Clinical Data

**Figure 5:**
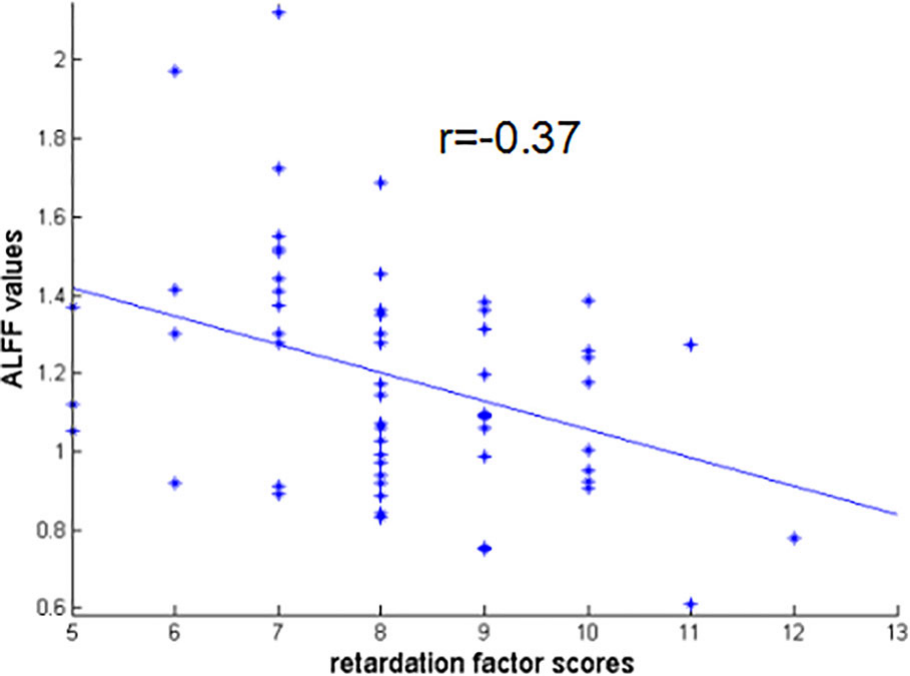
Negative correlation between the ALFF values of RO-MFG in AD group with retardation factor scores (r = –0.37, *p* = 0.003). ALFF, amplitude of low-frequency fluctuation; AD, anxious depression; RO-MFG, right orbital part of middle frontal gyrus.

There were negative correlations between the ALFF values of RO-MFG and both retardation factor scores and cognitive disturbance factor scores in AD group (r = -0.37, *p* = 0.003; r = -0.27, *p* = 0.035 uncorrected, respectively) (**Figure 5**). But the associations between the ALFF values of RO-MFG in AD group and cognitive disturbance factor scores no longer existed after multiple comparison testing.

### Functional Connectivity: Group Differences

No significant results were found among the three groups when using RO-MFG as seed ROI.

## Discussion

In this study, we used ALFF measurement to investigate the whole-brain activity in patients with AD. Our results showed that patients with AD had decreased ALFF values in RO-MFG when compared with UADs. Moreover, there is a strong negative correlation between the ALFF values of RO-MFG with retardation factor scores in AD patients. The above results supported our hypotheses that AD might have a reduced ALFF in the regions involved in automatic regulation of emotion.

In order to make a comprehensive analysis, we probed into the findings. The middle frontal gyrus, a region of the ventral part of the MPFC involved in processing emotion and automatic or implicit regulation of emotion (30, 31), is responsible for numerous cognitive functions, such as working memory (40), decision-making (41), attentional processing (42, 43), and top-down regulation in emotional processing (44). Therefore, the decreased ALFF values in RO-MFG, as well as its significant negative correlation with retardation factor scores, indicated an involvement of RO-MFG in the pathology of AD. Similar activity patterns had been found in emotion induction tasks study. Compared to UAD patients, AD patients showed lower activity in bilateral anterior lateral prefrontal cortices, which is also involved in top-down regulation in emotional processing (19). This finding in the present study is also supported by a large body of literatures linked to anxiety disorders and depressive disorders (36, 45–49). Wu et al. reported a decreased connectivity in MDD patients between right middle frontal gyrus and dorsal ACC (45), which is consistent with the results found in adolescent depression (46). Compared with the HCs, the MDD patients exhibited reduced fractional ALFF in the RO-MFG (47, 48). There were also reduced cortical volume of middle frontal gyrus and medial orbitofrontal gyrus in MDD patients (49). The decreased activity in RO-MFG could exhibit the combined side effects of reducing cognitive function, decreasing ability to use emotion adjustment strategies to modify amygdala response to fearful stimuli, leading to a weakening effect on mood regulation and increasing likelihood of suicide (50, 51). It is natural to infer that patients with AD would exhibit more severe depressive symptoms and a higher proportion of significant suicidal ideation and suicide attempts compared with UADs (6, 8, 22). Notwithstanding the studies in depressive disorder, the relevant literatures on anxiety disorders are also in accordance with our results (36, 52–55). Sylvester et al. summarized researches related to anxiety disorders, for example, generalized anxiety disorder (GAD) (52), panic disorder (53), posttraumatic stress disorder (PTSD) (54), and social anxiety disorder (SAD) (55), and found that the activity of the DMN declined in patients with anxiety disorder (36). However, there were some abnormalities in other brain regions in patients with anxiety disorders. For example, there was hyperactivation in left precuneus in both GAD and SAD (56, 57). Nevertheless, in patients with PTSD, the activity of the precuneus decreased after treatment (58). These findings illustrated that the difference in brain activity between AD and UAD is not entirely caused by anxiety.

In this study, we also found that the ALFF values in bilateral cerebellum crus2 in both AD patients and UAD patients were significantly lower than those in HC group. In addition, compared with HC group, decreased ALFF values in the right cerebellum crus1 in AD patients and decreased ALFF values in the left cerebellum crus1 were noted. Cerebellum crus1 and cerebellum crus2 are located in the posterior lobe of the cerebellum which is closely associated with cognitive and emotional functions (59, 60). A lot of MRI study of cerebellum in HC showed that cerebellum crus1and crus2 were related to cognitive control network (CCN) (60–63). Many fMRI studies in MDD found that cerebellum crus1 and crus2 showed decreased FC to the CCN, particularly to the DLPFC (64–66). Meta-analysis indicated that MDD was characterized by hypoconnectivity within CCN, so the ability of active regulation of emotion decreased (33). In summary, hypoactivity in cerebellum crus1and crus2 might be a potential biomarker of depression.

We also analyzed the limitations of our study for further improvements. Firstly, the population used for study was relatively small and consisted of only Chinese patients. Moreover, although the participants were asked to close their eyes and do not think during imaging, the participants still had thoughts or fell to sleep, which is inevitable. Last but not the least, given the absence of an anxiety control group in current study design, the study could not fully specify the effects of anxiety symptoms and depressive symptoms of AD on human brain activity. Future studies involving larger numbers of subjects with AD, UAD, anxiety disorder, and HC are needed to clarify the neural mechanisms behind AD.

In conclusion, the current study found that the patients with AD had impairment activity and connectivity in subcortical systems implicated in automatic regulation of emotion, including RO-MFG. The alterations in RO-MFG might play an important role in the symptomatology of AD.

## Data Availability Statement

All datasets generated for this study are included in the article/supplementary material.

## Ethics Statement

The studies involving human participants were reviewed and approved by Nanjing Brain Hospital, Medical School of Nanjing University. The patients/participants provided their written informed consent to participate in this study.

## Author Contributions

PZ conceptualized and designed the study. RY and XW analyzed the results and assisted in the writing of the study. JG was responsible for literature retrieval. MC proofread the article. QW contributed to data collection. QL and ZY revised the paper. All authors contributed and have approved the final manuscript.

## Funding

This work was supported by the National Natural Science Foundation of China (81871066, 81571639), Jiangsu Provincial Medical Innovation Team of the Project of Invigorating Health Care through Science, Technology and Education (CXTDC2016004), Jiangsu Provincial Key Research and Development Program (BE2018609).

## Conflict of Interest

The authors declare that the research was conducted in the absence of any commercial or financial relationships that could be construed as a potential conflict of interest.
